# Transjugular intrahepatic portosystemic shunt for the prevention of rebleeding in patients with cirrhosis and portal vein thrombosis: Systematic review and meta-analysis

**DOI:** 10.3389/fphar.2022.968988

**Published:** 2022-08-16

**Authors:** Ding-Fan Guo, Lin-Wei Fan, Qi Le, Cai-Bin Huang

**Affiliations:** ^1^ Department of Gastroenterology, The First Affiliated Hospital of Gannan Medical University, Ganzhou, China; ^2^ The First Clinical Medical School of Nanchang University, Nanchang, China; ^3^ Key Laboratory of Jiangxi Province for Transfusion Medicine, The First Affiliated Hospital of Nanchang University, Nanchang, China; ^4^ Department of General Surgery, The First Hospital of Lanzhou University, Lanzhou, China

**Keywords:** transjugular intrahepatic portosystemic shunt, liver cirrhosis, portal vein thrombosis, meta-analysis, systematic review

## Abstract

**Background:** Transjugular intrahepatic portosystemic shunt (TIPS) has been performed on patients with cirrhosis and portal vein thrombosis (PVT) to prevent rebleeding; however, the associated evidence is scarce. Hence, the study aimed to evaluate the feasibility and efficacy of TIPS in patients with cirrhosis and PVT and promote personalized treatment in such patients.

**Methods:** Literature was systematically obtained from PubMed, EMBASE, Cochrane Library, and Web of Science. Data from the included studies were extracted, and meta-analyses by the random effects model were used to pool data across studies. Heterogeneity was assessed using Cochran’s Q and I^2^ statistics. The source of heterogeneity was explored using subgroup analyses and meta-regressions.

**Results:** A total of 11 studies comprising 703 patients with cirrhosis and portal vein thrombosis (PVT: complete, 32.2%; chronic, 90.2%; superior mesenteric vein or splenic vein involvement, 55.2%; cavernous transformation, 26.8%) were included. TIPS showed feasibility in 95% of the cases (95% confidence interval [CI]: 89%–99%) with heterogeneity (I^2^ = 84%, *p* < 0.01) due to cavernous transformation. The pooled rebleeding rate was 13% (95% CI: 7%–20%) with heterogeneity (I^2^ = 75%, *p* < 0.01) explained by chronic PVT and anticoagulation (AC) therapy. Hepatic encephalopathy occurred in 32% of patients. The survival rate, portal vein recanalization rate, and shunt patency rate were 80%, 82%, and 77%, respectively.

**Conclusion:** TIPS is feasible and effectively prevents rebleeding in patients with cirrhosis and PVT, regardless of cavernous transformation of the portal vein. Due to a potentially high risk of rebleeding and no apparent benefits of AC, post-TIPS AC must be employed cautiously.

**Systematic Review Registration**: [https://www.crd.york.ac.uk/PROSPERO/display_record.php?RecordID=258765], identifier [CRD42021258765].

## Introduction

Non-neoplastic portal vein thrombosis (PVT) is a prevalent complication of liver cirrhosis, with incidence rates ranging from 10% to 23% (Qi et al., 2014; Harding et al., 2015). PVT raises portal vein pressure and reduces blood flow to the liver, worsening liver function, which is a hallmark of poor outcomes (Englesbe et al., 2010a; Werner et al., 2013). In addition, PVT increases mortality after liver transplantation and contraindicates the procedure when the thrombus extends to the superior mesenteric or splenic vein (Englesbe et al., 2010b). Variceal bleeding (VB) is a life-threatening complication with a 6-weeks mortality rate of 20% (Sarin et al., 2011). PVT increases the threat of VB and sometimes death by potentially increasing the portal vein pressure (Englesbe et al., 2010a).

At present, no consensus or guideline elucidates the optimal prophylactic treatment for patients with cirrhosis with VB and PVT. Standard treatments, including endoscopic treatments such as endoscopic band ligation and non-selective beta-blockers (NSBB), provide an effect to achieve immediate hemostasis and maximize the prevention of rebleeding (de Franchis, 2015). Transjugular intrahepatic portosystemic shunt (TIPS) is advised when patients fail to respond to endoscopic therapy and NSBB (de Franchis, 2015). Studies have reported that patients with cirrhosis and PVT require long time to achieve complete variceal eradication (Englesbe et al., 2010a; Dell'Era et al., 2014). Moreover, PVT may aggravate VB after endoscopic variceal ligation (Huang et al., 2020). Endoscopic treatments such as endoscopic band ligation are disconnection procedures that increase portal vein pressure. The side effects of NSBB treatment may lead to thrombus formation by reducing splanchnic blood flow (Groszmann et al., 2005). Hence, the standard treatments have limitations.

Recent advances in interventional radiological techniques and refinement of stent materials could facilitate the use of TIPS in complex cases, even with cavernous transformation of the portal vein (CTPV), which has been viewed as a contraindication in the past (Boyer and Haskal, 2010; Han et al., 2011). Studies have investigated the efficacy and safety of TIPS in patients with cirrhosis and PVT (Van Ha et al., 2006; Han et al., 2011; Luca et al., 2011; Luo et al., 2015; Wang et al., 2015; Lakhoo and Gaba, 2016; Qi et al., 2016; Wang et al., 2016; Luo et al., 2018; Lv et al., 2018; Lv et al., 2021). Two randomized clinical trials (RCT) reported that TIPS placement effectively prevented recurrent VB in patients with cirrhosis and PVT (Luo et al., 2015; Lv et al., 2018).

However, implementation of TIPS in a clinical setting is low due to the lack of consensus on the details of TIPS in preventing rebleeding in patients with cirrhosis and PVT. Hence, the present study conducted a systematic review and meta-analysis on the feasibility and efficacy of TIPS in preventing rebleeding in patients with cirrhosis and PVT to facilitate personalized treatment.

## Methods

This systematic review was conducted according to the Preferred Reporting Items for Systematic Reviews and Meta-Analyses statement and registered with PROSPERO (CRD 42021258765) (Page et al., 2021).

The following definitions were adopted in the study: Complete PVT was defined as an occlusion that occupied the entire crucial portal vein vessel lumen. Chronic PVT was defined as the presence of portal cavernoma, replacement of the original principal portal vein with a fibrotic cord, or a low intraluminal density on contrast-enhanced computed tomography (CT) (Lv et al., 2018). Post-TIPS anticoagulation (AC) was defined as a long-term AC (warfarin and other anticoagulant drugs for at least 6 months) after TIPS. Technical feasibility was defined as successful access to the portal vein, formation of an intrahepatic shunt between the hepatic and portal veins, and placement of stents. Recanalization was defined as the complete disappearance of the previous thrombosis.

### Search strategy

PubMed, Cochrane Library, EMBASE, and Web of Science were searched systematically from the inception to October 2021. Search terms, such as liver cirrhosis, hepatic fibrosis, and portal vein thrombosis, were devised for the population, whereas TIPS and transjugular intrahepatic portosystemic shunt were devised for the intervention. The medical subject heading, Embase subject heading, and free text terms were used to maximize search sensitivity.

### Study selection and data extraction

The inclusion and exclusion criteria were predefined to reduce the risk of bias. The inclusion criteria were as follows: 1) patients with cirrhosis and PVT diagnosed using imaging; 2) patients receiving TIPS to treat VB; and 3) reported rebleeding and clinical outcomes. The exclusion criteria were as follows: 1) letters, editorials, case reports, reviews, and animal experiments; 2) studies unavailable in English or Chinese; 3) patients with cancer or Budd–Chiari syndrome; 4) exclusively postoperative PVT; 5) follow-up period <6 months; and 6) insufficient outcome data. The articles with the highest number of cases or the most applicable information were selected in the case of studies with multiple publications. Two authors, D.F. Guo and L.W. Fan screened the titles and abstracts identified in the literature search and scrutinized the potentially eligible studies by reading full texts, extracting the following information: 1) Characteristics of the included studies and patient populations: first author, publication year, country, study design, and the number of patients, age, sex, etiology of cirrhosis, Child-Pugh classification, Model for end-stage liver disease score, and thrombosis characteristics; 2) characteristics of TIPS placement: indication for TIPS, approach to the portal vein, types of stents, related AC therapy, portosystemic pressure gradient (PPG) reduction, and additional procedures; and 3) clinical follow-up: the number and proportion of patients with rebleeding, hepatic encephalopathy (HE), survival, recanalization, shunt patency, and technical feasibility. Moreover, relevant information was obtained from the authors whose studies lacked critical information.

Most discrepancies in opinions were resolved through discussion between the two authors. If unresolved, the opinion of the third author (C.B. Huang) was sought.

### Quality assessment

D.F. Guo and L.W. Fan independently assessed all the included articles. The quality assessments for non-randomized and randomized studies were conducted using the risk of bias in non-randomized studies of interventions tool (Sterne et al., 2016) and the Cochrane risk of bias 2.0 tool (Sterne et al., 2019), respectively.

### Statistical analysis

A meta-analysis was performed to devise the pooled proportions and 95% confidence interval (CI), followed by Freeman–Tukey double arcsine transformation of the raw proportions (Barendregt et al., 2013). Assuming that heterogeneity was present among the participant studies, calculations were determined using the random effects model. Heterogeneity among studies was evaluated using the Cochran’s Q test (*p* < 0.1 was considered significant) and I^2^ statistic (values of 25%, 50%, and 75% indicated low, moderate, and high degrees of heterogeneity, respectively) (Higgins et al., 2003; Huedo-Medina et al., 2006). Potential factors associated with the heterogeneity were examined using subgroup analysis and a meta-regression model. Several predefined potential confounders were considered in the subgroup analysis and meta-regression model: study design, the proportions of complete and chronic PVT, CTPV, superior mesenteric vein (SMV), or splenic vein (SV) involvement, covered stent, approach to PV, and post‐TIPS AC. Publication bias was assessed using Egger’s linear regression test and funnel plot (the number of included studies was ≥10). Statistical analysis was performed using the R software (version 4.1.0; R Foundation Inc.; http://cran.r-project.org/).

## Results

### Study selection and quality assessment

**FIGURE 1:**
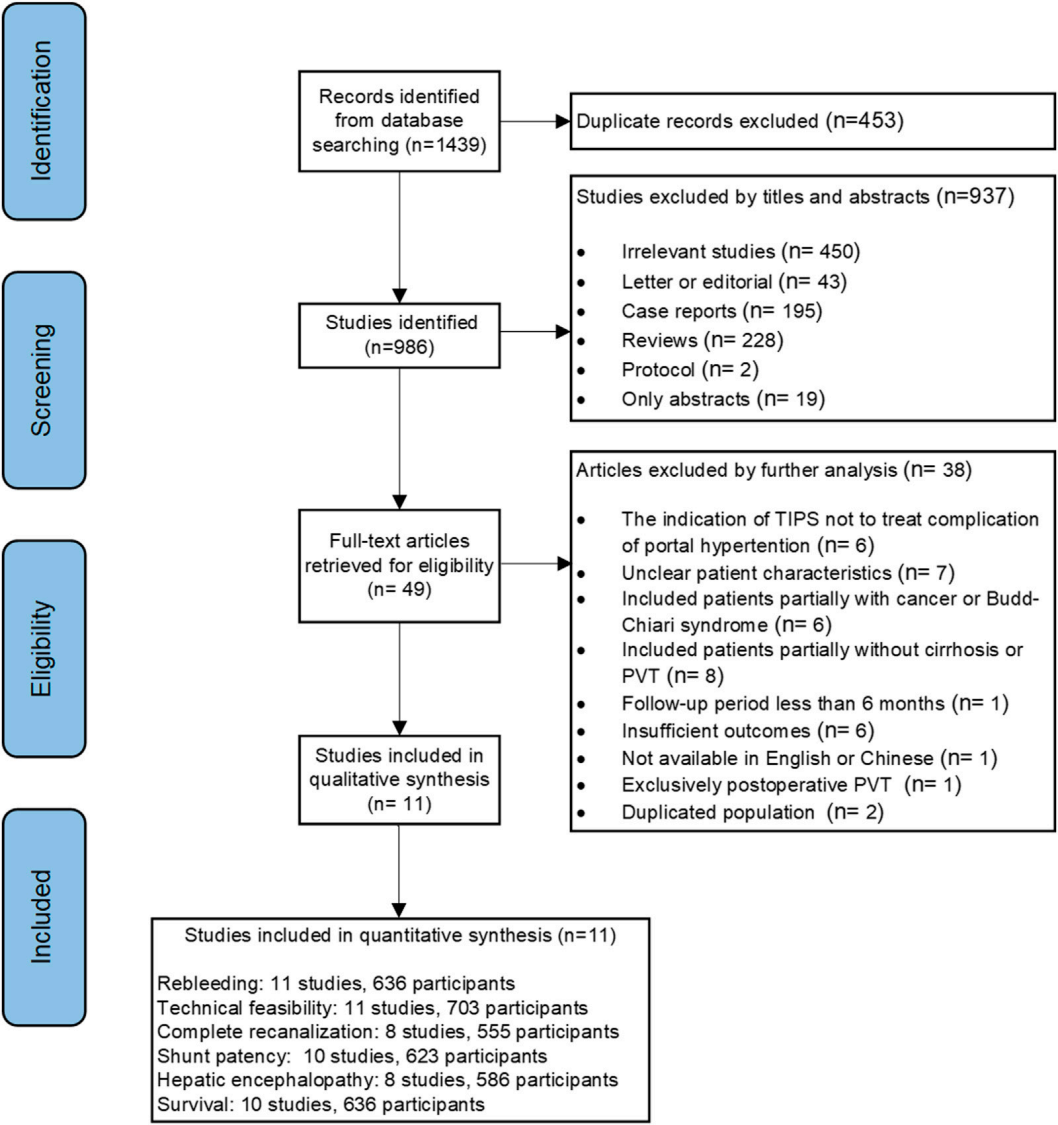
Flowchart showing the selection of studies for the present systematic review and meta-analysis.

After the initial search, 1,439 citations were retrieved from the database. After removing the duplicate results, 986 records were selected for screening. Of these, 11 full-text articles followed the predefined criteria and were included in the meta-analysis (Van Ha et al., 2006; Han et al., 2011; Luca et al., 2011; Luo et al., 2015; Wang et al., 2015; Lakhoo and Gaba, 2016; Qi et al., 2016; Wang et al., 2016; Luo et al., 2018; Lv et al., 2018; Lv et al., 2021). A flowchart illustrating the study selection process is shown in Figure 1. The quality of the studies was estimated, and the risk of bias is shown in Supplementary Figure S1.

### Characteristics of included studies and patients

As shown in Table 1, 11 studies (3 RCT and 8 observational studies) published over the last 15 years were included in the meta-analysis. Eight, two, and one studies were performed in China, America, and Italy, respectively. Many studies were excluded due to the inclusion of cancer or Budd–Chiari syndrome. Most studies reported that the leading etiology of cirrhosis is viral hepatitis. PVT was complete and chronic in 32.2% and 90.2% of cases, respectively, SMV or SV involvement was present in 55.2% of cases, and cavernous transformation was observed in 26.8% of cases.

**TABLE 1:** Characteristics of the included studies and patient populations.

Study	Country	Design	Patients number	Male (%)	Age	Etiology, viral/other	Child-pugh A/B/C	MELD score	Characteristics of PVT			
									Complete(%)	Chronic (%)	SMV or SV(%)	CTPV (%)
Lv et al. (2021)	China	Prospective	324	195 (60.2)	52.6	264/60	102/183/39	11.8	94/324 (29.0)	N/A	192/324 (59.3)	107/324 (33.0)
Luo et al. (2018)	China	Retrospective	24	19 (79.2)	44.6	17/7	7/14/3	10.7	24/24 (100.0)	24/24 (100.0)	3/24 (12.5)	N/A
Lv et al. (2018)	China	RCT	24	13 (54.2)	49.0^a^	21/3	9/13/2	12.0^a^	8/24 (33.3)	22/24 (91.7)	22/24 (91.7)	11/24 (45.8)
Wang et al. (2016)	China	RCT	64	38 (59.4)	54.8	53/11	24/32/8	10.8	N/A	61/64 (95.3)	24/64 (37.5)	4/64 (6.3)
Qi et al. (2016)	China	Prospective	51	31 (60.8)	51.5	35/16	8/34/9	8.1	23/51 (45.1)	N/A	N/A	24/51 (47.1)
Lakhoo and Gaba, (2016)	America	Retrospective	12	5 (41.7)	63.0^a^	3/9	4/5/3	15.0^a^	0/12 (0)	7/12 (58.3)	9/12 (75.0)	0/12 (0)
Wang et al. (2015)	China	Retrospective	25	22 (88.0)	47.3	22/3	3/20/2	12.0	2/25 (8.0)	25/25 (100.0)	4/25 (16.0)	0/25 (0)
Luo et al. (2015)	China	RCT	37	19 (51.4)	50.8	30/7	0/25/12	14.2	13/37 (35.1)	37/37 (100.0)	N/A	0/37 (0)
Luca et al. (2011)	Italy	Retrospective	70	47 (67.1)	55.0	43/27	17/42/11	11.6	24/70 (34.3)	52/70 (74.3)	52/70 (74.3)	2/70 (2.9)
Han et al. (2011)	China	Retrospective	57	20 (35.1)	50.0	40/17	25/26/6	N/A	14/57 (24.6)	57/57 (100.0)	N/A	30/57 (52.6)
Van Ha et al. (2006)	America	Retrospective	15	12 (80.0)	53.0^a^	N/A	0/11/4	18.3	4/15 (26.7)	11/15 (73.3)	2/15 (13.3)	4/15 (26.7)

MELD, Model for End-Stage Liver Disease; PVT, portal vein thrombosis; SMV, superior mesenteric vein; SV, splenic vein; CTPV, cavernous transformation of portal vein; RCT, randomized controlled trail; N/A, not accessible.

a Data was expressed as median.

### Application of TIPS technique and its technical feasibility

As shown in Table 2, the main indication for TIPS was portal hypertension, a complication of cirrhosis. Two studies reported an additional indication of maintaining the portal vein patency before liver transplantation. All studies included patients with VB. Other complications, such as refractory ascites and refractory hydrothorax, were also observed. In five studies, the traditional transjugular approach to the portal vein was used, and in one study, the transhepatic approach was used. In the other five studies, the transhepatic/transsplenic approach to the portal vein was used when the traditional transjugular approach failed. In nine studies, concomitant variceal embolization and local thrombolysis were employed. In two studies, the bare-metal stents to complete shunt creation were employed, whereas, in nine studies, covered stents were employed. Different covered stents, including the viatorr, fluency, and unspecified expanded polytetrafluoroethylene stent grafts, were employed in the studies. In seven studies, post-TIPS AC was used. Anticoagulant methods included oral warfarin and low-molecular-weight heparin. PPG reduction ranged from 10 to 19 mm Hg.

**TABLE 2:** Characteristics of TIPS placement.

Study	Indication for TIPS	Approach to PV	Additional procedure	Covered stents (%)	AC post-TIPS (%)	AC methods	PPG (mmHg)		
							Before TIPS	After TIPS	Reduction
Lv et al. (2021)	PH complication	TJ, TH, TS	Some used variceal embolization	285/285 (100.0)	197/285(69.1)	Oral warfarin	23.0	8.3	15.7
Luo et al. (2018)	PH complication	TH	Some used variceal embolization	22/22 (100.0)	21/22 (95.5)	LMWH, oral warfarin	22.0	10.6	11.4
Lv et al. (2018)	PH complication	TJ, TH, TS	5 used local thrombolysis, 7 used variceal embolization	23/23 (100.0)	21/23 (91.3)	LMWH, oral warfarin	27.7	8.7	19.0
Wang et al. (2016)	PH complication	TJ	some used mechanical lysis with a balloon catheter	64/64 (100.0)	31/64 (48.4)	LMWH, oral warfarin	21.2	9.8	11.4
Qi et al. (2016)	PH complication	TJ, TH, TS		26/43 (60.5)	0/43 (0)		N/A	N/A	N/A
Lakhoo and Gaba, (2016)	PH complication, PV patency pre-LT	TJ	3 used variceal embolization	12/12 (100.0)	0/12 (0)^a^		18.0	8.0	10.0
Wang et al. (2015)	PH complication	TJ	some used variceal embolization	25/25 (100.0)	25/25 (100.0)	Oral warfarin	20.4	9.1	11.3
Luo et al. (2015)	PH complication	TJ	21 used variceal embolization	37/37(100.0)	37/37 (100.0)	LMWH, oral warfarin	27.5	10.4	17.1
Luca et al. (2011)	PH complication, PV patency pre-LT	TJ	1 used variceal embolization	57/70 (81.4)	0/70 (0)		20.8	8.5	12.3
Han et al. (2011)	PH complication	TJ, TH, TS		0/43 (0)	43/43 (100.0)	LMWH, oral warfarin	25.7	14.0	11.7
Van Ha et al. (2006)	PH complication	TJ, TH	1 used local thrombolysis	0/13 (0)	0/13 (0)^a^		20.0	8.0	12.0

TIPS, transjugular intrahepatic portosystemic shunt; PV, portal vein; AC, anticoagulation; PPG, portosystemic pressure gradient; PH, portal hypertension; TJ, transjugular; TH, transhepatic; TS, transsplenic; LT, liver transplantation; LMWH, low-molecular-weight heparin; N/A, not accessible.

a Anticoagulant time was less than 6 months.

**FIGURE 2:**
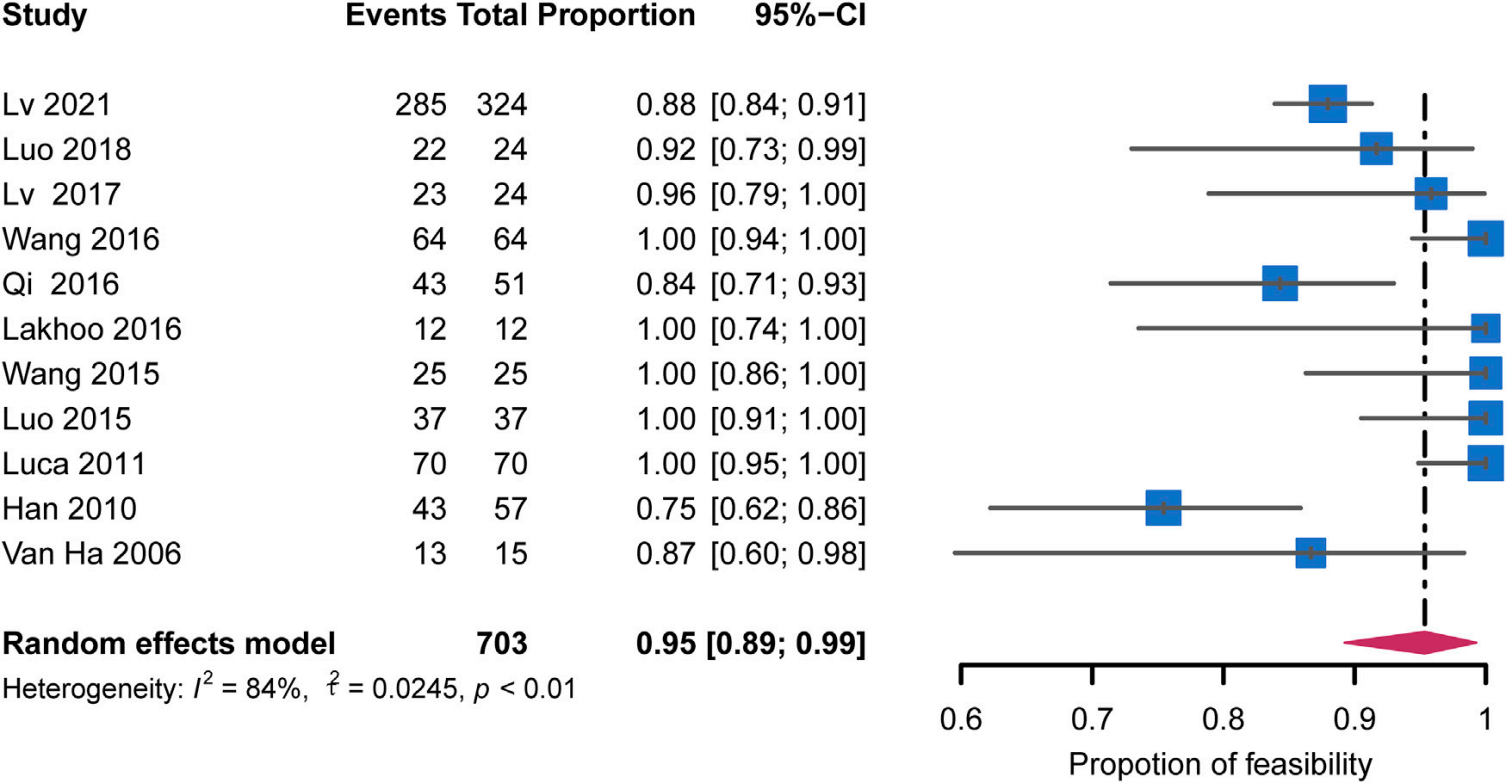
Forest plots for pooled rates of technical feasibility.

**FIGURE 3:**
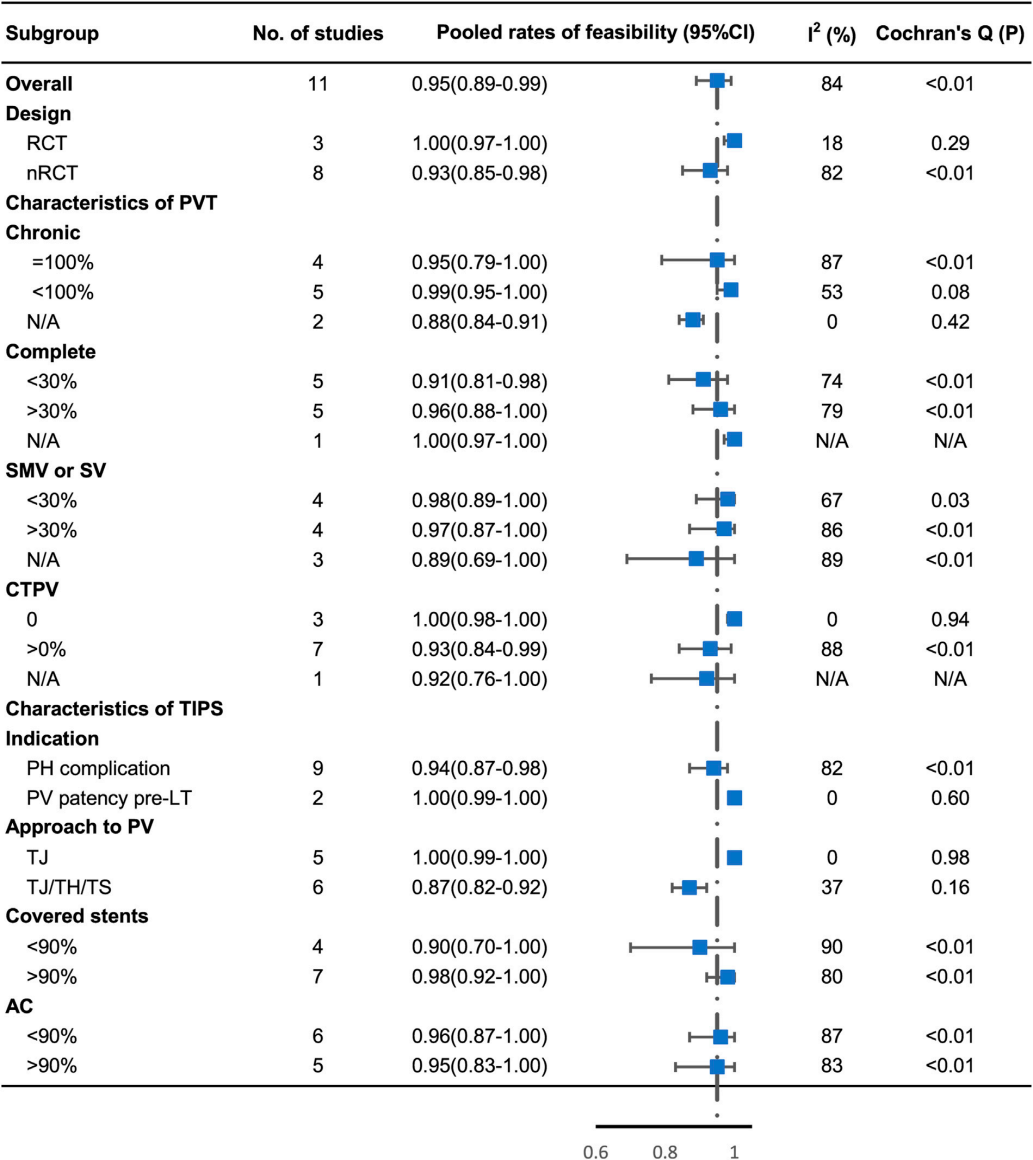
Subgroup analysis of technical feasibility by study design, proportion of complete and chronic PVT, proportion of CTPV, proportion of involvement of SMV or SV, indication of TIPS, approach to PV, proportion of covered stent, and proportion of post‐TIPS AC.

The forest plot showed the feasibility rate for each study and a pooled rate of 95% (95% CI: 89%–99%) with high heterogeneity (I^2^ = 84%, *p* < 0.01) (Figure 2). The rate in the studies that excluded patients with CTPV increased to 100% (95% CI: 98%–100%) without heterogeneity (I^2^ = 0%, *p* = 0.94). A similar result was found in five studies using only the transjugular approach, with the pooled rate of 100% (95% CI: 98%–100%) without heterogeneity (I^2^ = 0%, *p* = 0.98). The subgroup analyses are shown in Figure 3. Publication bias was not significant (Egger’s test, z = 1.02, *p* = 0.33) (Supplementary Figure S2A).

### Rebleeding

**FIGURE 4:**
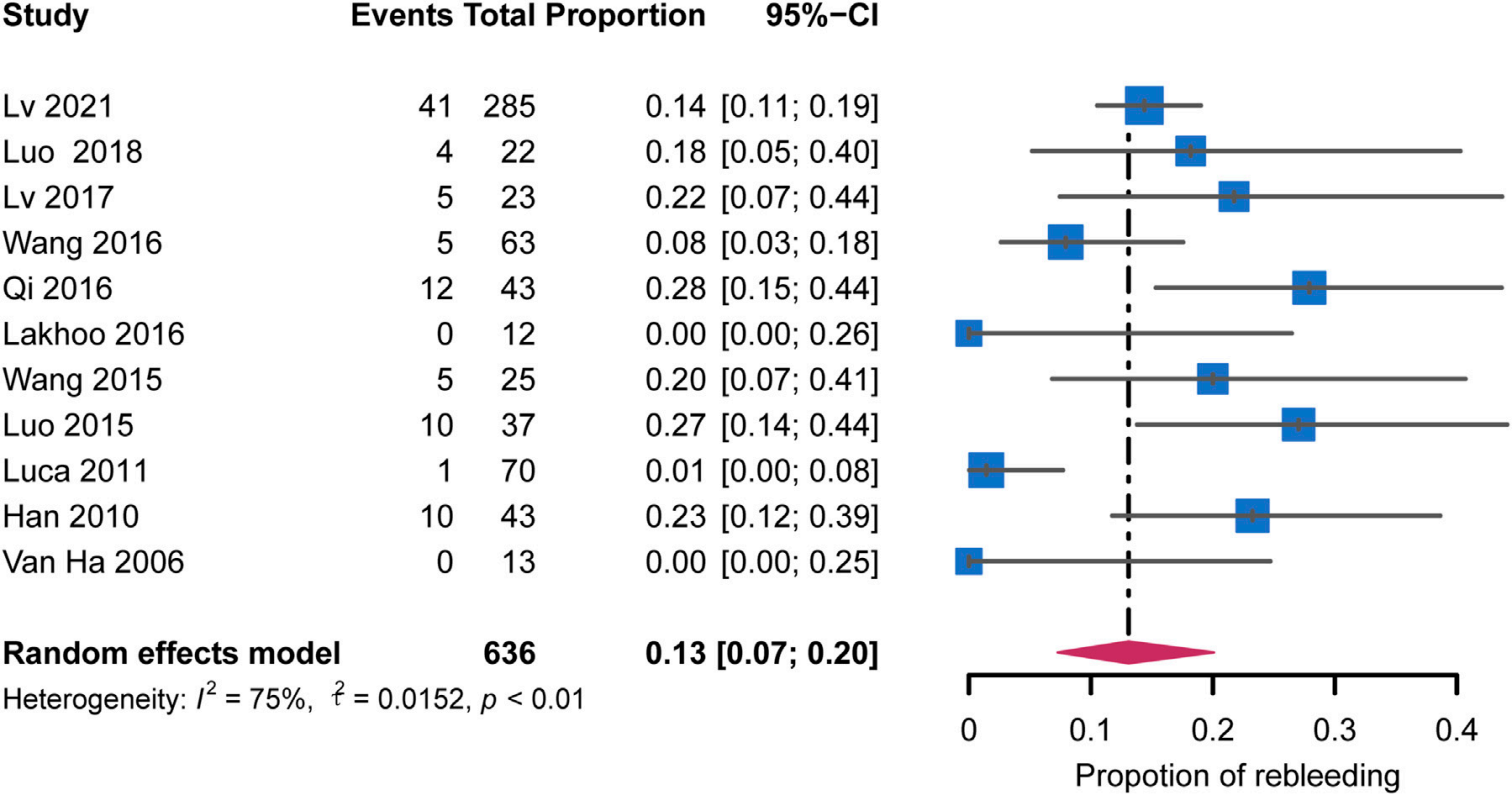
Forest plots for pooled rates of rebleeding.

**FIGURE 5:**
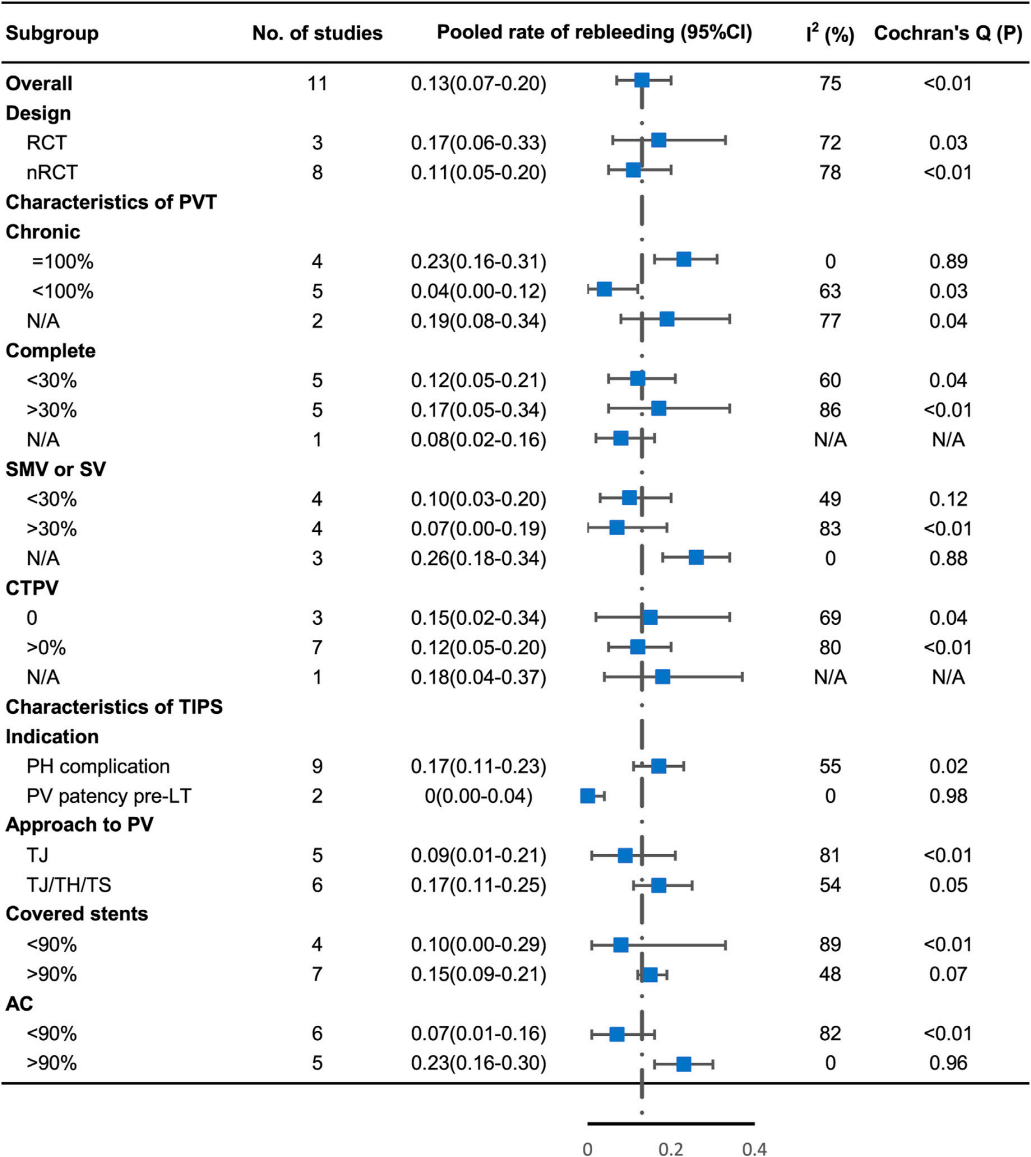
Subgroup analysis of rebleeding by study design, proportion of complete and chronic PVT, proportion of CTPV, proportion of involvement of SMV or SV, indication of TIPS, approach to PV, proportion of covered stent, and proportion of post‐TIPS AC.

All studies reported the overall rebleeding rate ranging from 0% to 27.9% (Table 3). The pooled rebleeding rate was 13% (95% CI: 7%–20%) with high heterogeneity (I^2^ = 75%, *p* < 0.01) (Figure 4). The rebleeding rate in four studies that exclusively included patients with chronic PVT increased to 23% (95% CI: 16%–31%) without heterogeneity (I^2^ = 0%, *p* = 0.89). Studies that involved not less than 90% of patients receiving AC showed an elevated pooled rebleeding rate of 23%. No heterogeneity was observed in this subgroup (I^2^ = 0%, *p* = 0.96). Other factors associated with the heterogeneity were not confirmed. The subgroup analyses are shown in Figure 5. Funnel plot and Egger’s test (z = 0.07, *p* = 0.94) showed no significant publication bias (Supplementary Figure S2B).

**TABLE 3:** Clinical follow-up.

Study	Technical feasibility (%)	Rebleeding (%)	HE (%)	Survival (%)	Complete recanalization (%)	Shunt patency (%)	Follow-up time (months)
Lv et al. (2021)	285/324 (88.0)	41/285 (14.4)	82/285(14.0)	210/285 (73.7)	267/285 (93.7)	217/285 (76.1)	>6.0
Luo et al. (2018)	22/24 (91.7)	4/22 (18.2)	4/22 (18.2)	19/22 (86.4)	N/A	17/22 (77.3)	34.0
Lv et al. (2018)	23/24 (95.8)	5/23 (21.7)	6/23 (26.1)	15/23 (65.2)	19/22 (86.4)	19/22 (86.4)	30.9^a^
Wang et al. (2016)	64/64 (100.0)	5/63 (7.9)	13/63 (20.6)	62/63 (98.4)	49/63 (77.8)	58/63 (92.1)	12.0
Qi et al. (2016)	43/51 (84.3)	12/43 (27.9)	26/43 (60.5)	27/43 (62.8)	N/A	32/43 (74.4)	40.1^a^
Lakhoo and Gaba, (2016)	12/12 (100.0)	0/12 (0)	N/A	9/12 (75.0)	7/12 (58.3)	N/A	15.0^a^
Wang et al. (2015)	25/25 (100.0)	5/25 (20.0)	N/A	20/25 (80.0)	20/23 (87.0)	20/25 (80.0)	25.6
Luo et al. (2015)	37/37 (100.0)	10/37 (27.0)	15/37 (40.5)	25/37 (67.6)	24/37 (64.9)	25/37 (67.6)	22.8
Luca et al. (2011)	70/70 (100)	1/70 (1.4)	22/70 (31.4)	60/70 (85.7)	40/70 (57.1)	43/70 (61.4)	23.4^a^
Han et al. (2011)	43/57 (75.5)	10/43 (23.3)	13/43 (30.2)	35/43 (81.4)	43/43 (100.0)	26/43 (60.5)	>6.0
Van Ha et al. (2006)	13/15 (86.7)	0/13 (0)	N/A	11/13 (84.6)	N/A	12/13 (92.3)	17.0^a^

HE, hepatic encephalopathy; N/A, not accessible.

a Data was expressed as median.

### Hepatic encephalopathy and survival

**FIGURE 6:**
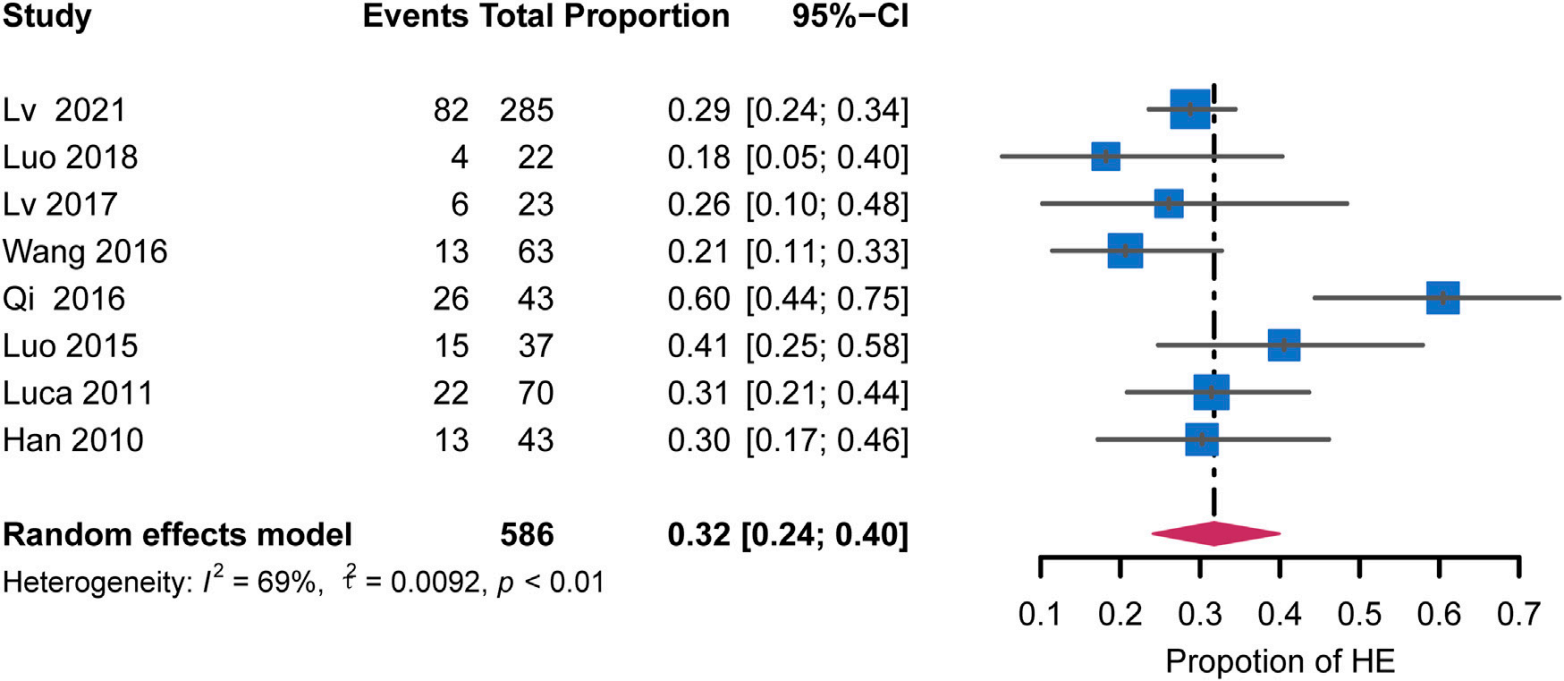
Forest plots for pooled rates of HE.

HE incidence was reported in 8 of 11 studies and varied among studies, ranging from 14% to 60.5% (Table 3). The pooled rate was 32% (95% CI: 24%–42%) with moderate heterogeneity (I^2^ = 69%, *p* < 0.01) (Figure 6). The remaining studies, excluding those that included patients with chronic PVT, showed an HE incidence of 26% (95% CI: 19%–33%) with no heterogeneity (I^2^ = 0%, *p* = 0.38). Regarding the extent of PVT, the HE incidence was lower in studies that included 30% of patients with SMV or SV than that in the remaining studies (20% vs. 29%, respectively). Heterogeneity among these studies was not significant (I^2^ = 0%, *p* = 0.87). The HE incidence was 30% (95% CI: 22%–39%) in studies using post-TIPS AC with low heterogeneity (I^2^ = 10%, *p* = 0.34). These subgroup analyses are shown in Supplementary Figure S3. The publication bias was not examined due to the low number of studies.

**FIGURE 7:**
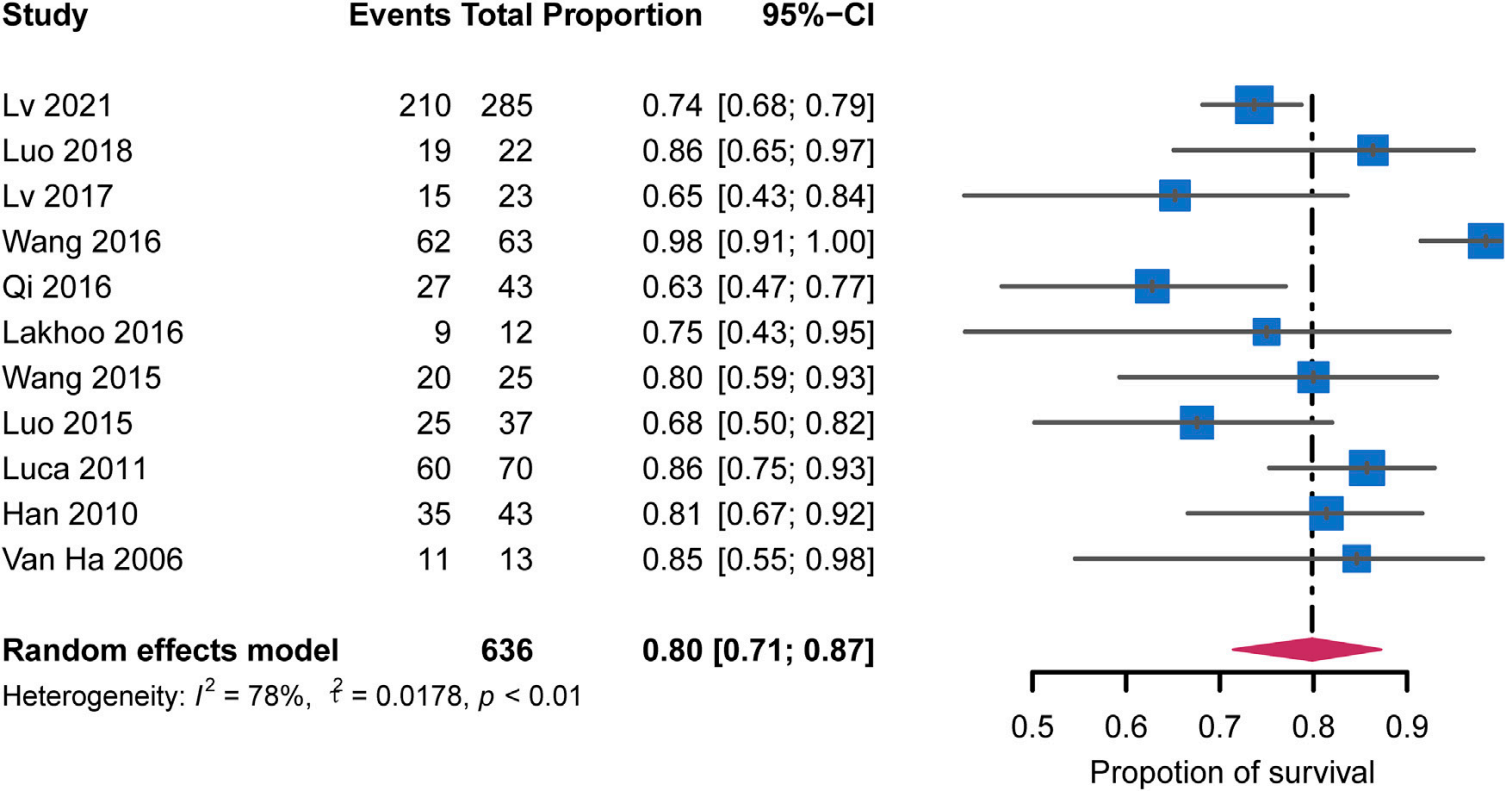
Forest plots for pooled rates of survival.

All 11 studies were included in the meta-analysis of survival rates. The pooled survival rate for all studies was 80% (95% CI: 71%–87%) with high heterogeneity (I^2^ = 78%, *p* < 0.01) (Figure 7). In the studies using post-TIPS AC, the survival rate was similar (76%; 95% CI: 68%–84%) with low heterogeneity (I^2^ = 19%, *p* = 0.31). These subgroup analyses are shown in Supplementary Figure S4. Egger’s test (z = 0.30, *p* = 0.77) showed no significant publication bias (Supplementary Figure S2C).

### Portal vein recanalization and shunt patency

Eight studies reported data on complete recanalization. The recanalization rate ranged from 57.1 to 93.7%, with a pooled rate of 82% (95% CI: 67%–93%; I^2^ = 92%) (Supplementary Figure S5). The recanalization rate of studies involving at most 30% of patients with SMV or SV was 81% (95% CI: 71%–88%). Heterogeneity among these studies was not significant (I^2^ = 0%, *p* = 0.38). The subgroup analysis is shown in Supplementary Figure S6. The publication bias was not estimated since the number of studies was <10.

Shunt patency rates were reported in 10 studies, and the pooled rate was 77% (95% CI: 69%–83%; I^2^ = 63%) (Supplementary Figure S7). Egger’s test (z = 0.22, *p* = 0.83) showed no significant publication bias (Supplementary Figure S2D).

### Subgroup analysis

The studies were divided into subgroups based on the distribution of observed characteristics, as shown in Figures 3, 5. All other subgroup analysis results, which have been discussed above, are provided in the Supplementary Figures.

### Meta-regression analysis

Meta-regression was performed on technical feasibility, rebleeding, and survival. As shown in Table 4, a transhepatic/transsplenic approach to the portal vein was significantly associated with a decreased technical feasibility rate. In contrast, AC therapy post-TIPS was significantly associated with a higher rebleeding rate. Due to insufficient data, the meta-regression analysis was not conducted for other factors and outcomes.

**TABLE 4:** Meta-regression analysis according to outcomes.

Factors	Feasiblity			Rebleeding			Survival		
	Coeff.	95%CI	P	Coeff.	95%CI	P	Coeff.	95%CI	P
Study characteristics									
RCT vs. non-RCT	0.176	−0.153 to 1.402	0.098	0.075	−0.136 to 0.286	0.489	0.046	−0.163 to 0.254	0.667
TIPS technical and treatment characteristics									
Approach to PV	−0.302	−0.383 to −0.222	<0.001	0.114	−0.063 to 0.292	0.206	−0.113	−0.287 to 0.060	0.200
Covered or bare metal stents	0.155	−0.082 to 0.393	0.200	0.058	−0.140 to 0.255	0.567	0.015	−0.198 to 0.228	0.891
Post-TIPS AC	−0.020	−0.249 to 0.208	0.861	0.198	0.035 to 0.360	0.017	−0.069	−0.270 to 0.133	0.506

Coeff., coefficient; HE, hepatic encephalopathy; PV, portal vein; TIPS, transjugular intrahepatic portosystemic shunt; RCT, randomised controlled trail; AC, anticoagulation.

## Discussion

The present systematic review and meta-analysis comprehensively and strictly examined three RCT and eight non-RCT studies to evaluate the feasibility and efficacy of TIPS in preventing rebleeding in patients with cirrhosis and PVT. The pooled analyses revealed that TIPS implantation was significantly feasible in most cases (95%). Regarding the clinical outcome, the pooled rebleeding rate was 13%, HE incidence was 32%, survival rate was 80%, recanalization rate was 82%, and shunt patency rate was 77%. These results showed that TIPS was significantly associated with effective prevention of rebleeding and high survival rate.

A previous meta-analysis of 12 studies designed to investigate the outcome of TIPS in patients with cirrhosis and PVT suggested that portal hypertension-associated complications are indications for TIPS (Zhang et al., 2021). The present study had some limitations. First, the low number of records available through database search showed that a comprehensive literature search was not performed, potentially producing biased results. Second, high heterogeneity in several analyses may have hindered the robust conclusions and recommendations. Unfortunately, the potential sources of heterogeneity and rebleeding-related clinical outcomes were not identified and discussed. Hence, further investigation is warranted to estimate the real benefit of TIPS before its widespread application.

First, we evaluated the technical feasibility of TIPS. CTPV was the main barrier hindering the implementation of TIPS. However, the feasibility rate decreased slightly from 95% to 93%, indicating that TIPS remained successful despite the presence of cavernous transformation. The subgroup analyses suggested that these patients had good outcomes after TIPS. Surprisingly, compared with the studies that used the traditional transjugular approach, advanced puncture techniques such as the transsplenic and transhepatic approaches did not improve the feasibility rate, perhaps since the two studies that employed the transsplenic and transhepatic approaches were published 10 years ago. Therefore, the feasibility could be improved with increased technical experience.

One of the key findings of this meta-analysis is that post-TIPS AC treatment is not necessary for certain patients with cirrhosis and PVT. Although post‐TIPS AC promoted recanalization in the subgroup analysis, it was associated with a higher rebleeding rate and a lower survival rate. Previous studies concluded that TIPS alone effectively maintained the portal vein patency due to the high-velocity flow created by the shunt, not requiring AC treatment (Wang et al., 2016; Rodrigues et al., 2019). In addition, heterogeneity prevailed after subgroup analyses based on the AC treatment, indicating that AC is not a unique source of heterogeneity. A slightly lower shunt patency rate was observed in the subgroup analysis when the thrombus extended to the SMV or SV. Hence, the extent of PVT should be considered to balance the risk of rebleeding and portal vein patency in the long-term clinical management of patients.

Another important aspect of our findings is that the covered stents for TIPS reduce HE incidence without decreasing the risk of rebleeding. Despite clinical heterogeneity, these results are crucial and may help advocate for covered stents (Bureau et al., 2007; Perarnau et al., 2014). A randomized multileft study stated the superiority of 8-mm stents in decreasing the rate of spontaneous overt HE and severe and recurrent/persistent HE after TIPS (Wang et al., 2017). Further, the size of covered stents is essential. However, this meta-analysis showed that the post-TIPS HE incidence was high, a major post-TIPS complication yet (Han et al., 2011). In addition, improved shunt patency and recanalization were observed in the subgroup analyses based on the type of stent but with heterogeneity. This implies that recanalization and shunt patency are associated with the characteristics of PVT. The specific sources of heterogeneity were not found, as shunt patency and recanalization are dependent on multiple factors, including patient characteristics, stent sizes, types of stents, and operator expertise (Patidar et al., 2014; Tang et al., 2017).

Although TIPS and its associated materials and stents have been developed and refined over the last two decades, the placement of TIPS remains a rescue or second-line therapy in patients with cirrhosis and PVT. TIPS is performed when AC treatment is contraindicated or in patients with uncontrolled bleeding post endoscopic therapy. Therefore, there is a low utilization rate in the actual clinical setting, with only some patients with cirrhosis receiving the TIPS placement. Nevertheless, it is encouraging that TIPS has significant clinical benefits and may provide novel insights into treating patients with cirrhosis and PVT. Further studies are warranted to accumulate sufficient evidence to standardize operating procedures, associated adjuvant drug treatments, and periprocedural care, optimizing current treatment strategies.

The present systematic review and meta-analysis have a few shortcomings. First, technical methods, types, and manufacturers of stents might bring significant heterogeneity to this meta-analysis; however, these factors were not considered. Nevertheless, most heterogeneity could be explained using subgroup analysis with a random-effects model and meta-regression. Second, the included studies spanned 15 years during which the techniques and medical devices for TIPS have made swift advances. Nevertheless, the endpoints of previous and current studies were homogeneous; thus, these studies were included in the quantitative studies. Third, many related studies were excluded from our meta-analysis due to the lack of outcomes and full-text availability. However, an extensive search strategy was performed to collect all related information, and no evidence of publication bias was revealed. Lastly, a difference in the follow-up time was observed among the included studies, limiting the interpretation of some outcomes.

## Conclusion

TIPS is feasible and effectively prevents rebleeding in patients with cirrhosis and PVT, including those with CTPV. Due to a potentially high risk of rebleeding and no apparent benefits of AC, post-TIPS AC must be used cautiously. Further, the characteristics of PVT should be considered before making decisions on the TIPS procedure and during long-term clinical management.

## Data Availability

The original contributions presented in the study are included in the article/Supplementary Material, further inquiries can be directed to the corresponding author.

## Abbreviations

AC, Anticoagulation; CTPV, Cavernous transformation of the portal vein; CI, Confidence interval; HE, Hepatic encephalopathy; MELD, Model for end-stage liver disease; NSBBs, Non-selective beta-blockers; PVT, Portal vein thrombosis; RCTs, Randomized clinical trials; LT, Liver transplantation; LWMH, Low-molecular-weight heparin; PV, Portal vein; PPG, Portosystemic pressure gradient; PH, Portal hypertension; SMV, Superior mesenteric vein; SV, Splenic vein; VB, Variceal bleeding; TJ, Transjugular; TH, Transhepatic; TS, Transsplenic; TIPS, Transjugular intrahepatic portosystemic shunts.
