# Supplementary material for: Transjugular intrahepatic portosystemic shunt for the prevention of rebleeding in patients with cirrhosis and portal vein thrombosis: Systematic review and meta-analysis
Source: Front Pharmacol. 2022 Aug 16;13:968988. doi: 10.3389/fphar.2022.968988 (PMC9424732; doi:10.3389/fphar.2022.968988)
Supplement: Supplementary file 1 [file Presentation1.zip › #_Suplementary figure legend.docx]

**Figure legend**

**Supplementary Figure 1** Risk of bias assessment for randomized (RoB 2.0) and non-randomized (ROBINS-I) studies.

**Supplementary Figure 2** Funnel plot - (a) feasibility (b) rebleeding (c) survival (d) shunt patency

**Supplementary Figure 3** Subgroup analysis of HE by study design, proportion of complete and chronic PVT, proportion of CTPV, proportion of involvement of SMV or SV, indication of TIPS, approach to PV, proportion of covered stent, and proportion of post‐TIPS AC

**Supplementary Figure 4** Subgroup analysis of survival by study design, proportion of complete and chronic PVT, proportion of CTPV, proportion of involvement of SMV or SV, indication of TIPS, approach to PV, proportion of covered stent, and proportion of post‐TIPS AC

**Supplementary Figure 5** Forest plots for pooled rates of complete recanalization

**Supplementary Figure 6** Subgroup analysis of recanalization by study design, proportion of complete and chronic PVT, proportion of CTPV, proportion of involvement of SMV or SV, indication of TIPS, approach to PV, proportion of covered stent, and proportion of post‐TIPS AC

**Supplementary Figure 7** Forest plots for pooled rates of TIPS patency

**Supplementary Figure 8** Subgroup analysis of shunt patency by study design, proportion of complete and chronic PVT, proportion of CTPV, proportion of involvement of SMV or SV, indication of TIPS, approach to PV, proportion of covered stent, and proportion of post‐TIPS AC

**Supplementary Figure 9** Subgroup analysis of rebleeding by portal pressure reduction.

**Supplementary Figure 10** Subgroup analysis of clinical outcomings by study size; (A) technical feasibility, (B) rebleeding, (C) HE, (D) survival, (E) recanalization, (F) shunt patency.
