# Supplementary figures and images for: Transjugular intrahepatic portosystemic shunt for the prevention of rebleeding in patients with cirrhosis and portal vein thrombosis: Systematic review and meta-analysis
Source: Front Pharmacol. 2022 Aug 16;13:968988. doi: 10.3389/fphar.2022.968988 (PMC9424732; doi:10.3389/fphar.2022.968988)

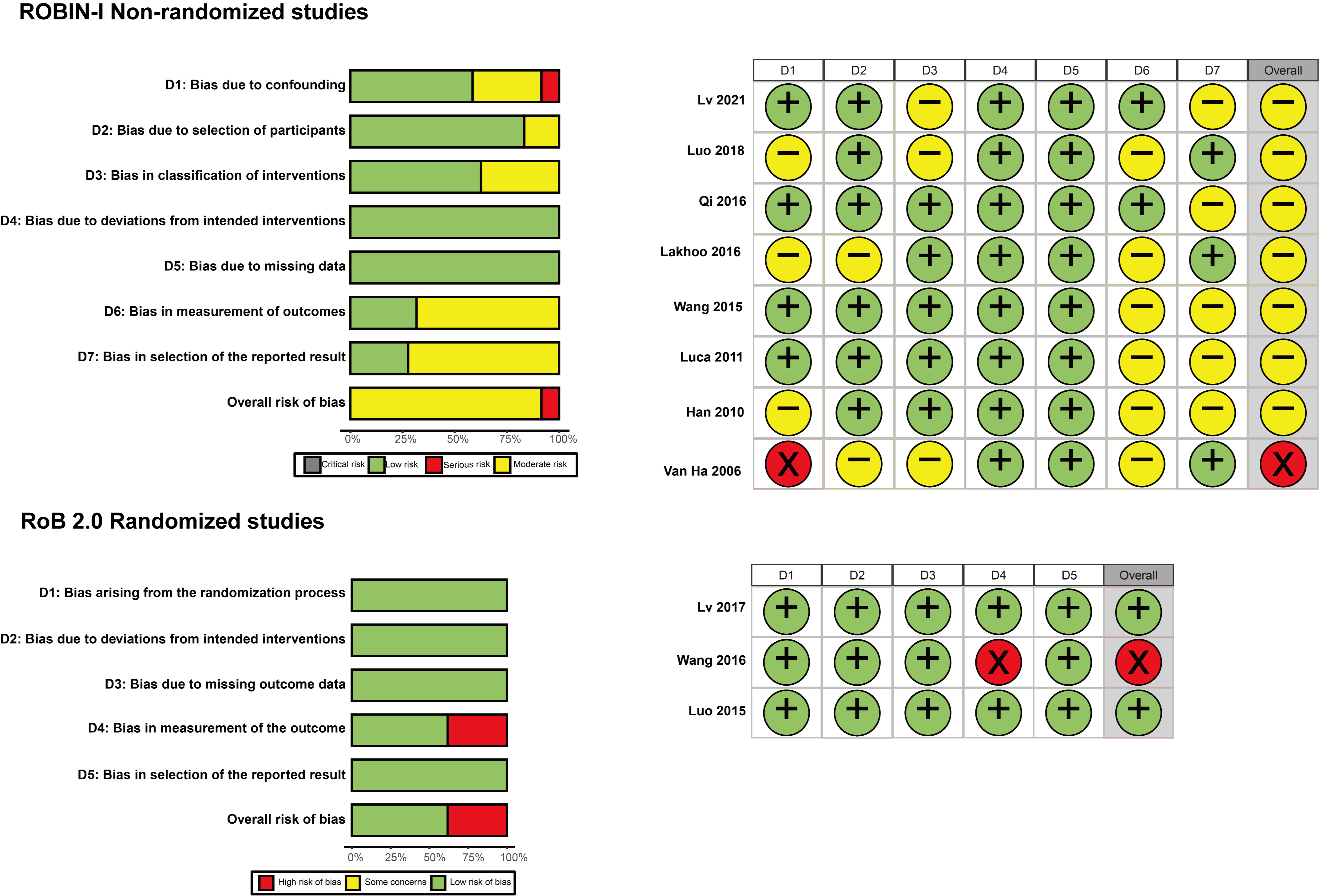

Supplement: Supplementary file 1 [file Presentation1.zip › Supplementary Figure 1 Risk of bias assessment.tif]

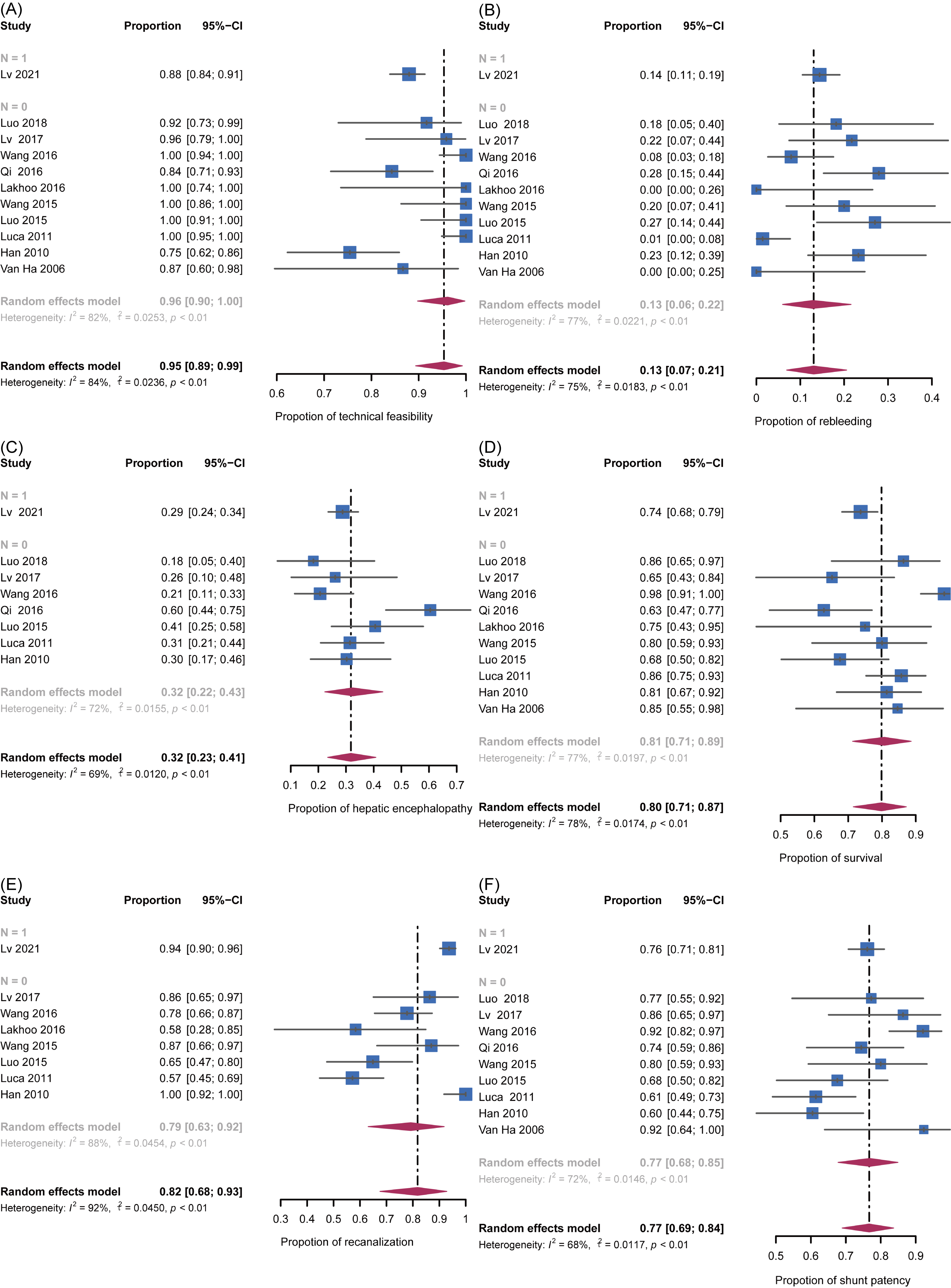

Supplement: Supplementary file 1 [file Presentation1.zip › Supplementary Figure 10.tif]

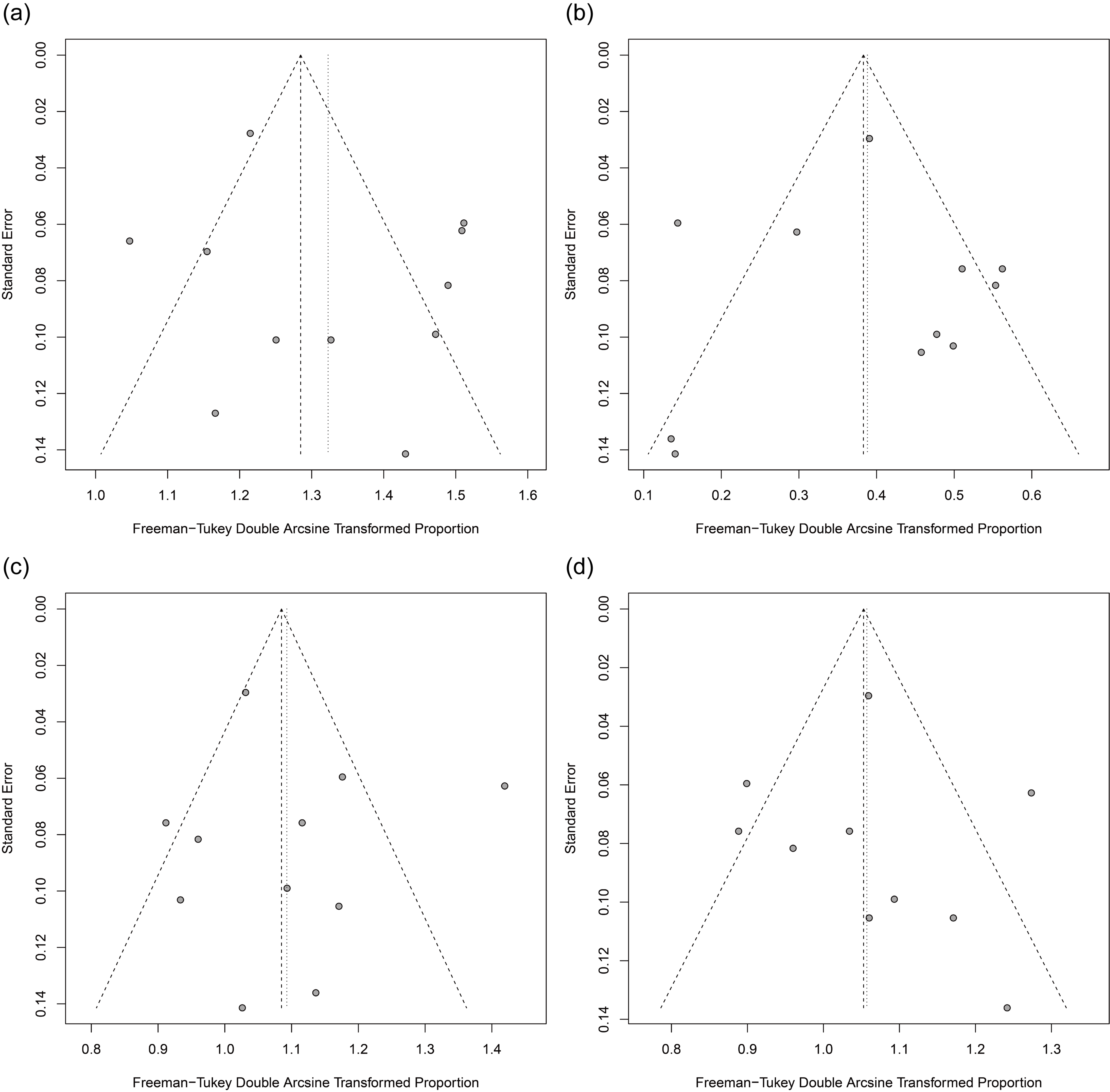

Supplement: Supplementary file 1 [file Presentation1.zip › Supplementary Figure 2 Funnel plot.tif]

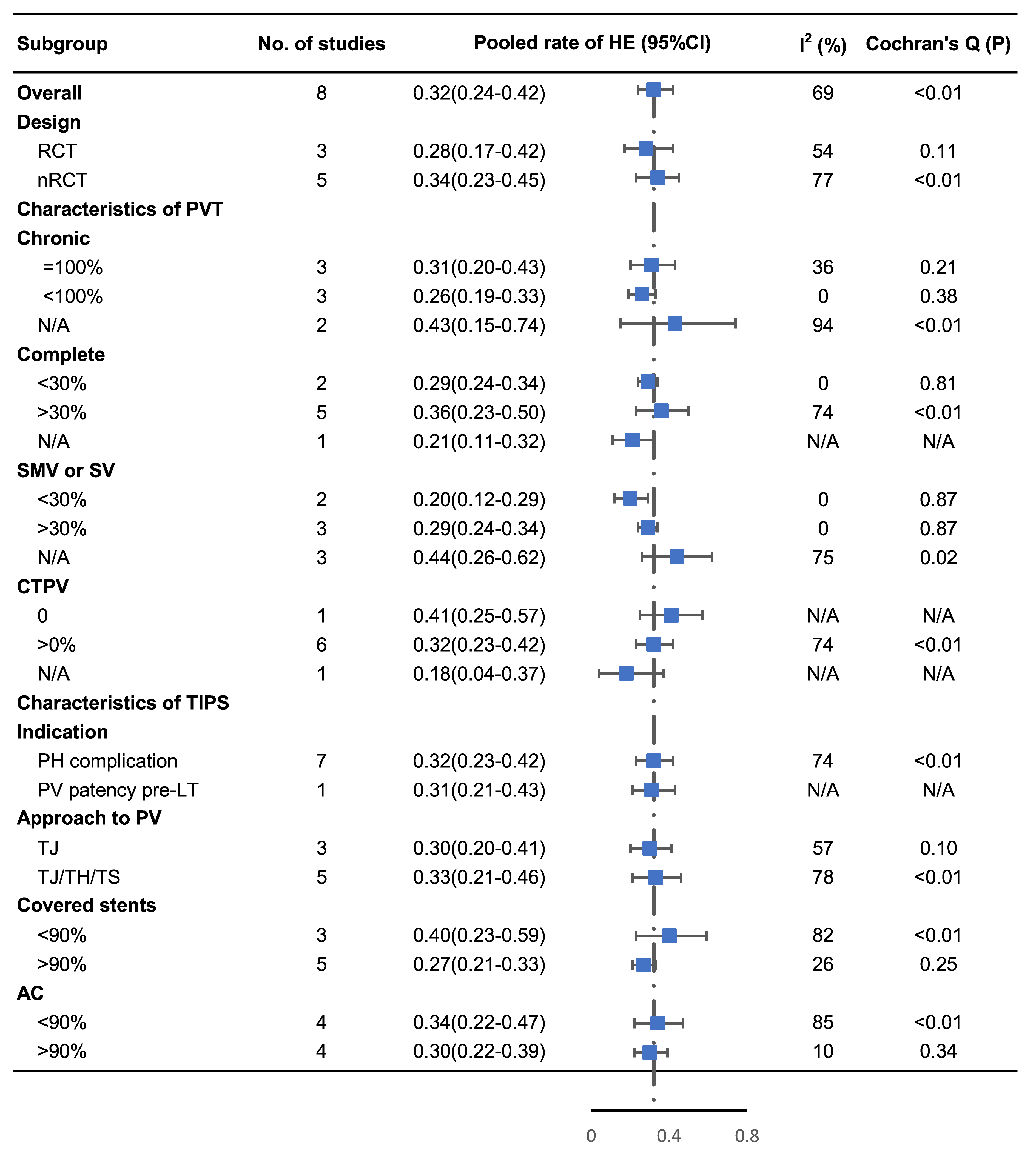

Supplement: Supplementary file 1 [file Presentation1.zip › Supplementary Figure 3 Subgroup analysis of HE.tif]

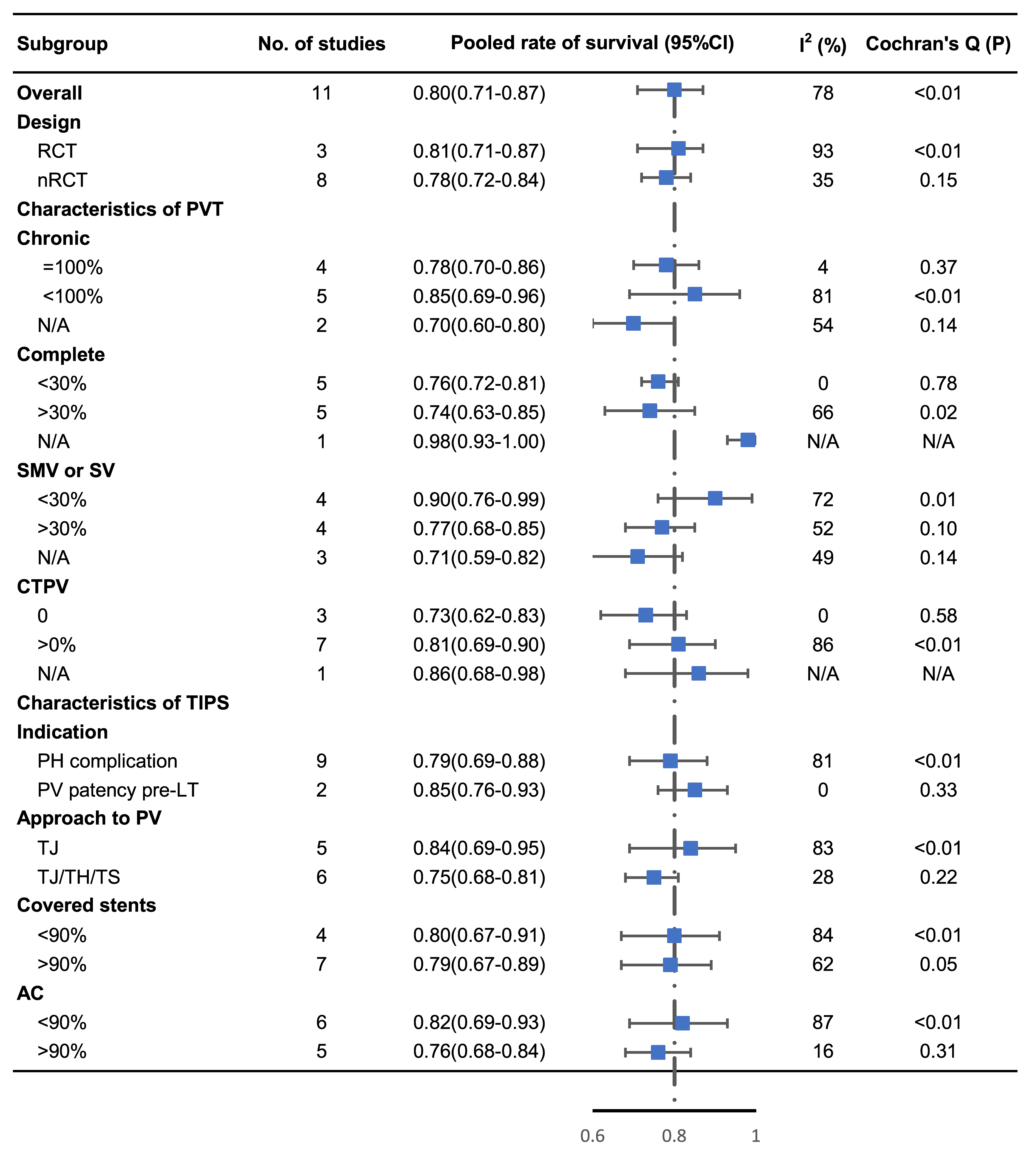

Supplement: Supplementary file 1 [file Presentation1.zip › Supplementary Figure 4 Subgroup analysis of survival.tif]

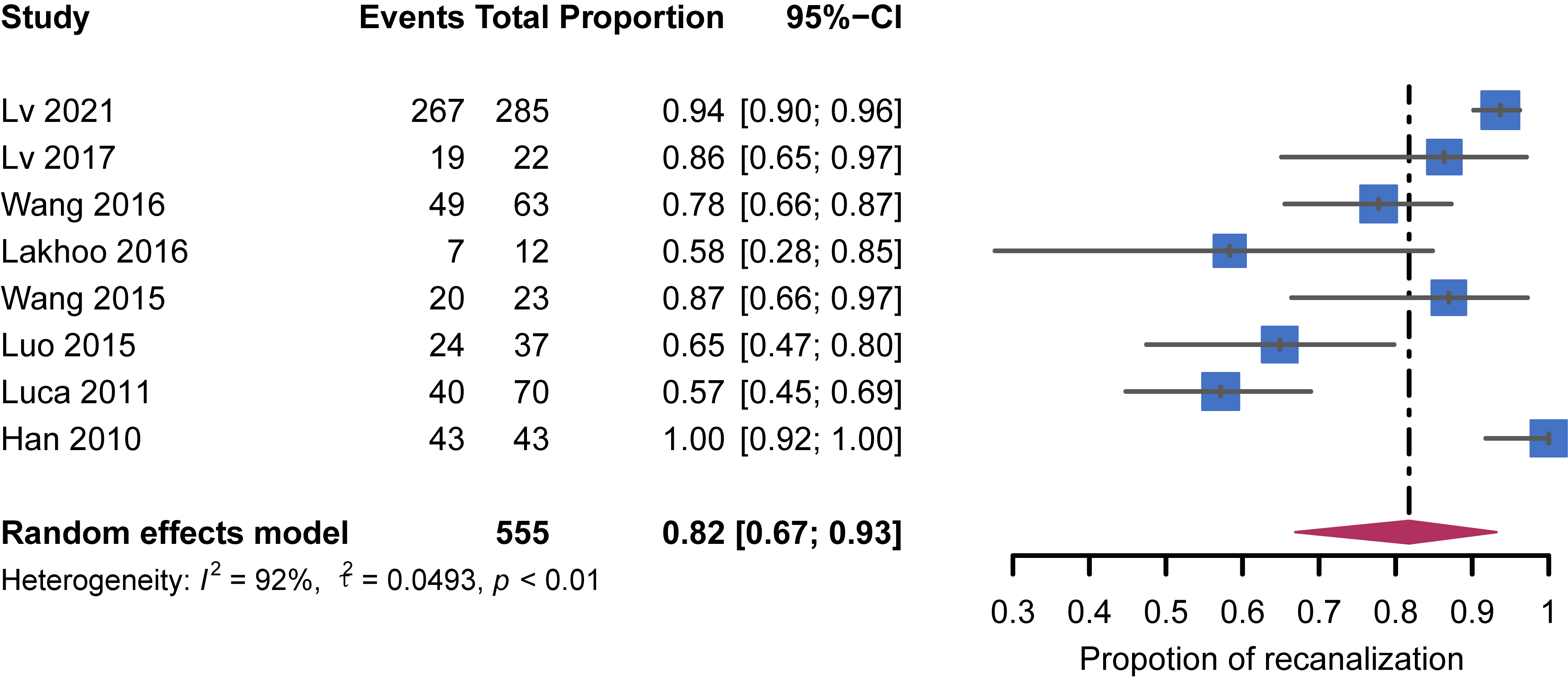

Supplement: Supplementary file 1 [file Presentation1.zip › Supplementary Figure 5 rates of complete recanalization.tif]

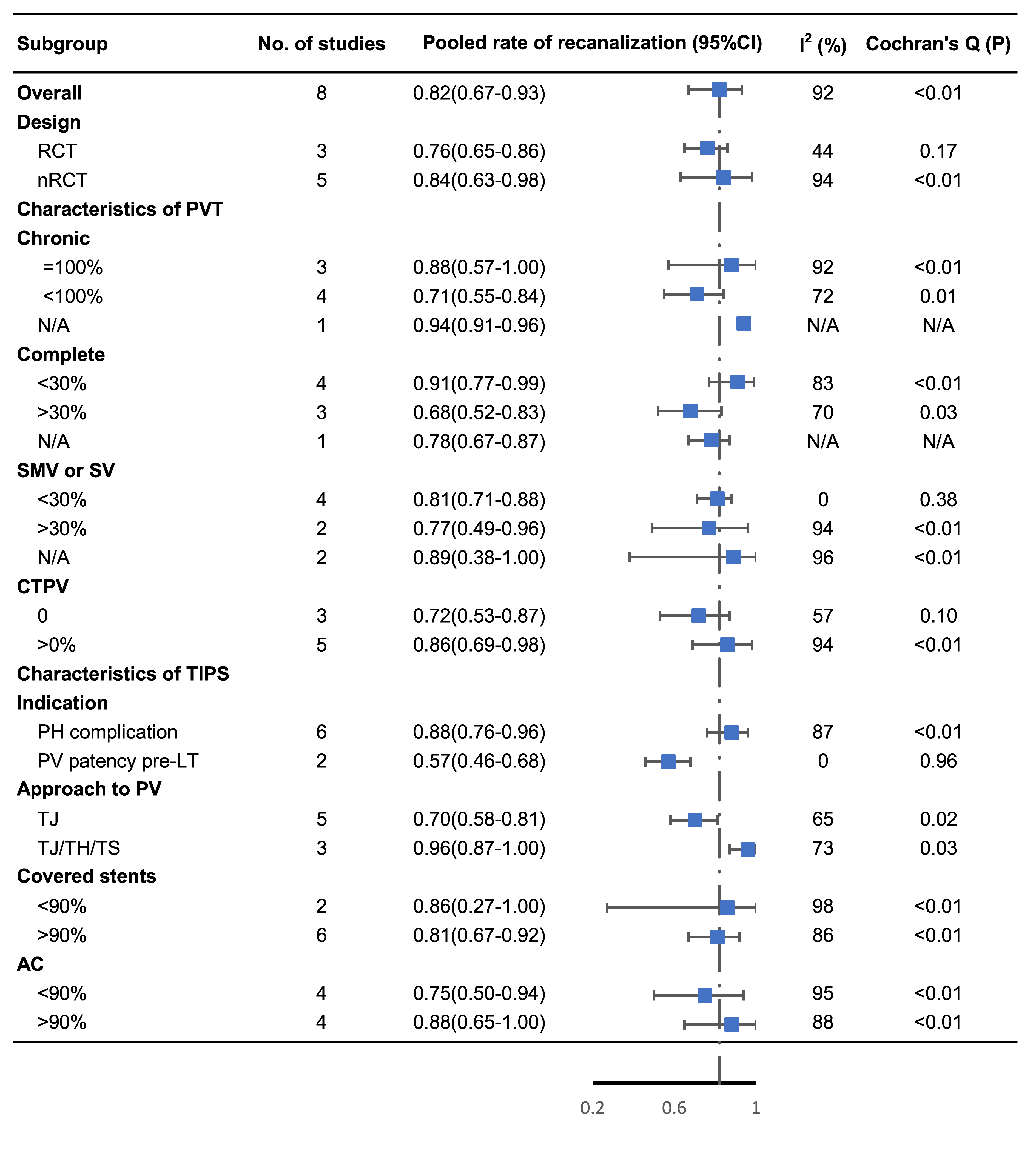

Supplement: Supplementary file 1 [file Presentation1.zip › Supplementary Figure 6 Subgroup analysis of recanalization.tif]

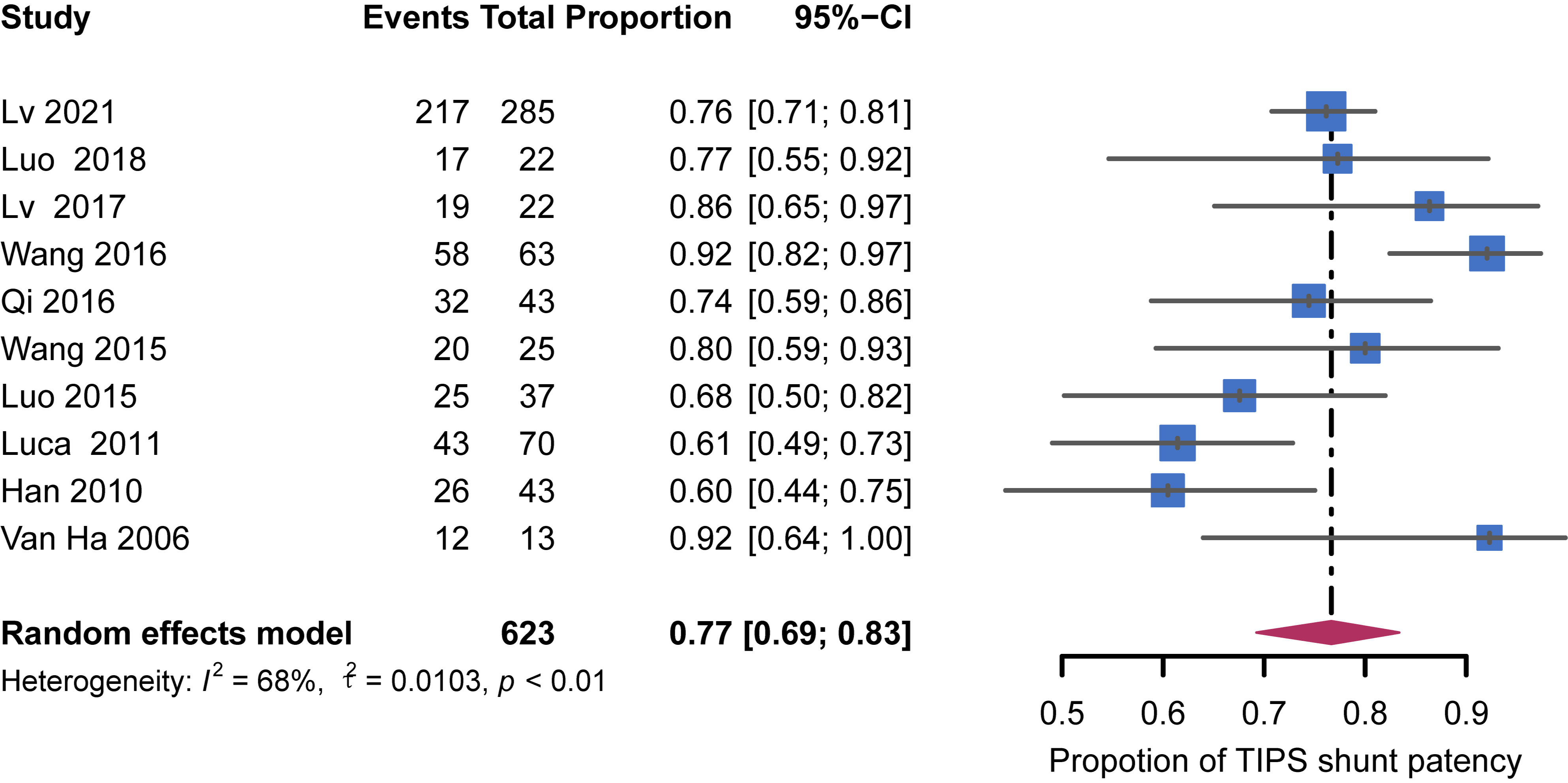

Supplement: Supplementary file 1 [file Presentation1.zip › Supplementary Figure 7 pooled rates of TIPS patency.tif]

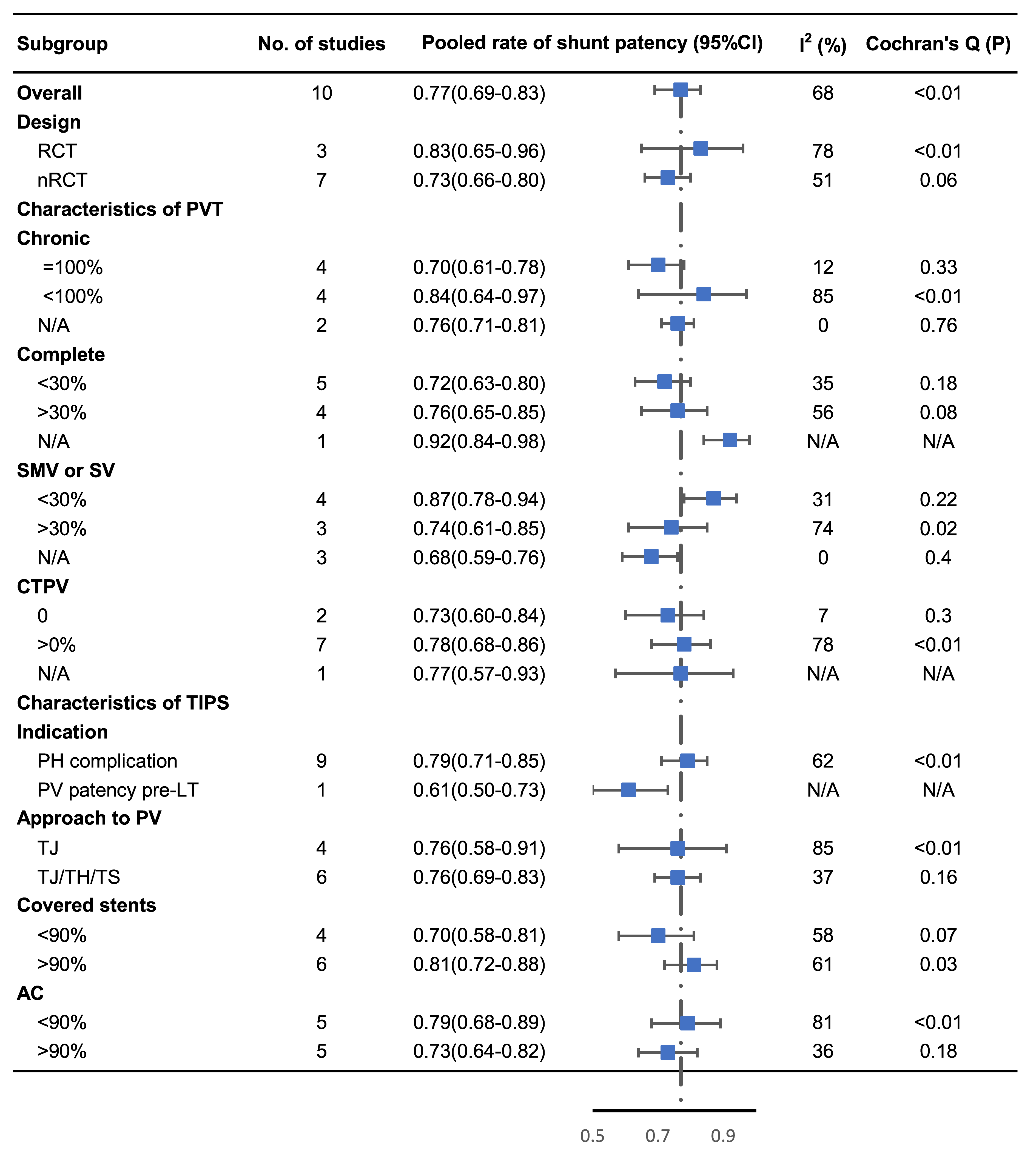

Supplement: Supplementary file 1 [file Presentation1.zip › Supplementary Figure 8 Subgroup analysis of shunt patency.tif]

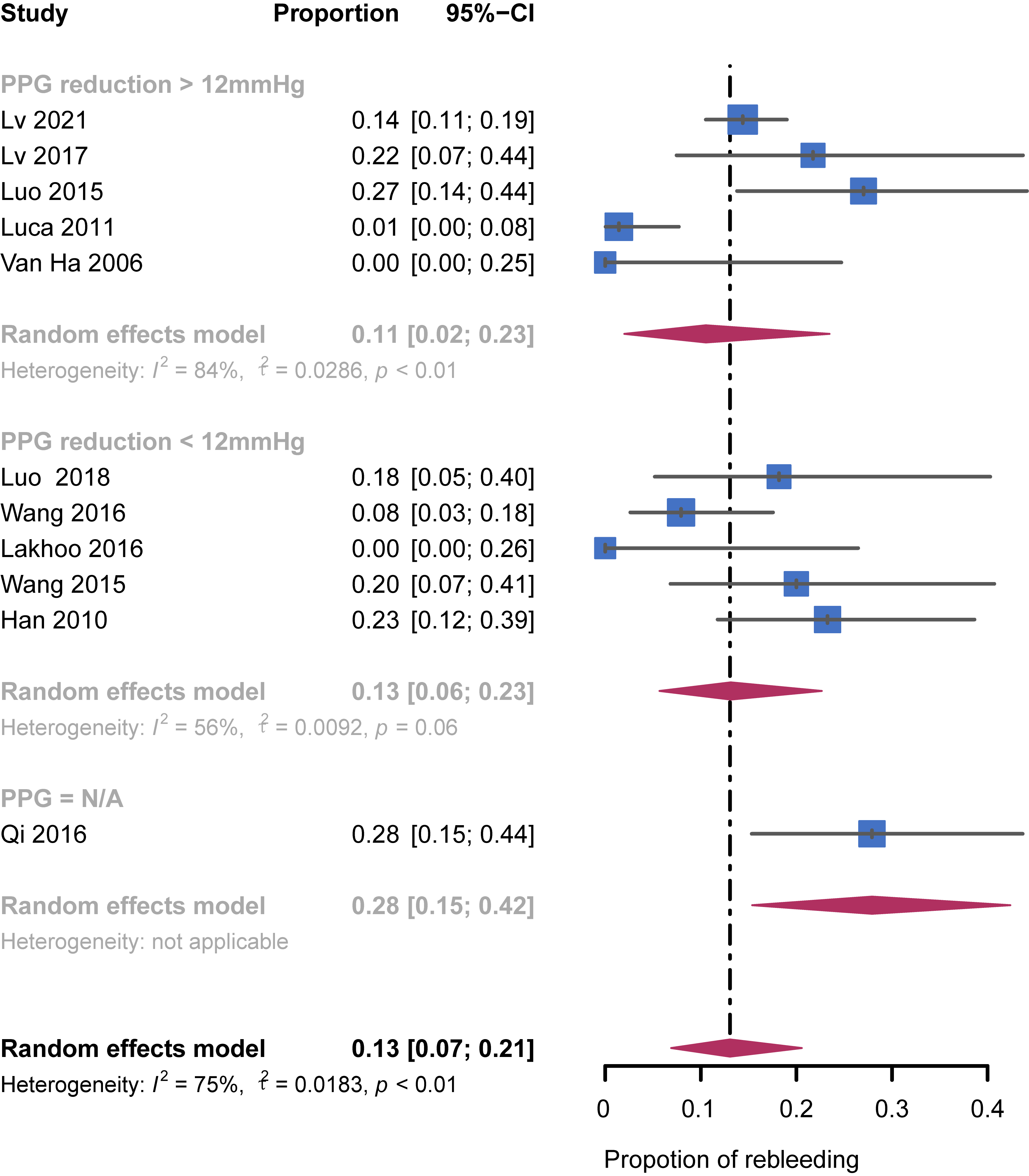

Supplement: Supplementary file 1 [file Presentation1.zip › Supplementary Figure 9.tif]
